# An Injectable Glass Polyalkenoate Cement Engineered for Fracture Fixation and Stabilization

**DOI:** 10.3390/jfb8030025

**Published:** 2017-07-05

**Authors:** Basel A. Khader, Sean A. F. Peel, Mark R. Towler

**Affiliations:** 1 Department of Mechanical Engineering, Faculty of Engineering and Architectural Science, Ryerson University, 350 Victoria Street, Toronto, ON M5B 2K3, Canada; basel.khader@ryerson.ca; 2 Keenan Research Centre, St. Michael’s Hospital, Toronto, ON M5B 1T8, Canada; 3 Division of Oral & Maxillofacial Surgery & Anaesthesia, Faculty of Dentistry, University of Toronto, Toronto, ON M5G 1G6, Canada; Sean.Peel@dentistry.utoronto.ca

**Keywords:** fracture fixation, distal radius fracture, germanium oxide, polyacrylic acid, injectable glass polyalkenoate cements, bovine serum albumin

## Abstract

Glass polyalkenoate cements (GPCs) have potential as bio-adhesives due to their ease of application, appropriate mechanical properties, radiopacity and chemical adhesion to bone. Aluminium (Al)-free GPCs have been discussed in the literature, but have proven difficult to balance injectability with mechanical integrity. For example, zinc-based, Al-free GPCs reported compressive strengths of 63 MPa, but set in under 2 min. Here, the authors design injectable GPCs (IGPCs) based on zinc-containing, Al-free silicate compositions containing GeO_2_, substituted for ZnO at 3% increments through the series. The setting reactions, injectability and mechanical properties of these GPCs were evaluated using both a hand-mix (h) technique, using a spatula for sample preparation and application and an injection (i) technique, using a 16-gauge needle, post mixing, for application. GPCs ability to act as a carrier for bovine serum albumin (BSA) was also evaluated. Germanium (Ge) and BSA containing IGPCs were produced and reported to have working times between 26 and 44 min and setting times between 37 and 55 min; the extended handling properties being as a result of less Ge. The incorporation of BSA into the cement had no effect on the handling and mechanical properties, but the latter were found to have increased compression strength with the addition of Ge from between 27 and 37 MPa after 30 days maturation.

## 1. Introduction

Minimally invasive procedures are receiving increasing attention in the field of orthopedics. Such procedures utilize injectable cements, usually, but not always, based on either calcium phosphate or acrylic cement chemistry. These materials can be molded to replicate the bone void and will then set in situ. Injectable systems offer many advantages over conventional bone cements and fillers, such as a reduction in the time it takes to perform the surgery alongside reductions in large muscle retraction, scar size and post-surgical pain [1]. Contemporary orthopedic implants often require the surgeon to drill out the surgical site around the implant, in turn causing increased bone loss, damage of adjacent tissues and extended surgical time, but minimally invasive procedures can ameliorate these issues [2].

Calcium phosphate cements (CPCs) were discovered by Brown and Chow in the early 1980s and were originally used for treating dental caries [3]. The first CPCs were based on tetracalcium phosphate (TTCP) and dicalcium phosphate anhydrous (DCPA), which, when combined, transform into HA at neutral pH under isothermic conditions and at physiologic pH [4]. Due to this, CPCs are osteoconductive and osseointegrative [3,5] but exhibit long setting times and insufficient mechanical properties for most load bearing applications, particularly in tension and shear [3]. However, they are now employed as bone substitutes in non-load bearing applications [3]. For example, an in vitro study investigated the stability of fracture fragments following fixation using CPCs, with and without the use of supplemental K-wires for treating unstable fractures of the distal end of the radius; they also examined fracture fragments following alternative techniques of percutaneous fracture fixation [6]. It was concluded that CPCs, on their own, were not sufficient to endure the physiologic flexion-extension movements of the wrist. Supplemental wire fixation (K-wires) with CPCs offered more stability than K-wires alone; however, the combination was found to offer considerably less stability than augmented external fixation [6]. The authors suggested that when CPC is used for treating unstable distal radial fractures then it is best to use supplemental fixation. A second study was conducted that examined the utility of CPC95 (Biopex; Mitsubishi Material, Saitama, Japan), an injectable CPC consisting of α-tricalcium phosphate (75 weight percent (wt %), tetracalcium phosphate (18 wt %), and dicalcium phosphate dihydrate (5 wt %) in treating fractures of the distal radius [7]. Seven patients experiencing distal radius fractures underwent closed reduction; two were fixed with external fixation while the remaining five were fixed with percutaneous pinning [7]. A 1 cm incision was made, exposing the fracture. An 11-gauge needle (Jamshidi disposable biopsy/aspiration needle; Baxter Healthcare, Deerfield, IL, USA) was then inserted and CPC95 was injected into the fracture site [7]. Patients were placed in a short arm cast following the procedure to prevent mobility of the wrist. It was found that CPC95 had a higher compressive strength (σc) (80–90 MPa) than cancellous bone (1.9–7.0 MPa) and a lower σc than cortical bone (89–164 MPa) [8,9]. The range of motion for the augmented wrists averaged 56° of extension, 45° of flexion, 19° of radial deviation, 36° of ulnar deviation, 84° of supination and 74° of pronation. Patient grip strength averaged 20.6 kg, 93% of that of the untreated hand [7]. One patient experienced mild stiffness in the fingers, while another reported asymptomatic nonunion of the ulnar styloid [7]. Extrusion of the injected CPC95 into the extensor tendon sheath was discovered in six patients; CPC95 was spontaneously resorbed in five of those patients within six months, while surgical removal of the cement was necessary for one patient at six months following surgery [7]. CPC95 has the potential to be used in treating distal radius fractures due to reports of early mobilization and functional recovery of the treated wrist [7]. Unfortunately, CPCs display a lack of cohesion due to disintegration upon early contact with blood [10]. Additionally, CPCs suffer from long setting times and insufficient mechanical properties for load bearing applications particularly in tensile and shear [3].

Glass polyalkenoate cements (GPCs) were developed in the early 1970s [11] in the Laboratory of the Government Chemist (London, UK). GPCs have properties suitable for orthopedic applications as they chemically bond to bone [12,13]. The chemical bond occurs via ion exchange at the interface between the GPC and the mineral phase of bone. GPCs set by the reaction between an aqueous solution of polyalkenoic acid (PAA) and an ion-leachable alumino-silicate glass; once mixed, the acid attacks the network modifiers in the glass network, releasing metal cations that cross-link the polyanion chains of the acid, subsequently resulting in a cement containing both reacted and unreacted glass particles [14]. GPCs have, to date, been restricted to dental [15] and ear, nose and throat (ENT) applications [16,17] due to the presence of aluminium (Al) in the glass phase of all commercial GPCs [4]. However, attempts have been made to tailor Al-free versions of these materials for fracture fixation. For example, titanium (Ti) has been substituted for silicon in the glass phase of some experimental GPCs as Ti has excellent biocompatibility and also acts as a former and modifier in the glass structure [18]. Zinc and silver ions have been added to the glass phase of GPCs because of their antimicrobial activity [19], and strontium (Sr) ions have been substituted for calcium (Ca) ions in order to increase radiopacity of the cement and stimulate bone formation around the implantation site [20,21,22]. Germanium (Ge) has been incorporated into the glass as it adopts the role of a network former and is theoretically able to isomorphically replace Si in the network [23]. The work contained herein expands partly on the authors’ earlier work [24], by further investigating the applicability of germano-silicate glasses as the glass component in an injectable GPC for fracture fixation whilst also evaluating the suitability of these materials as carriers for biologics; in this case, bovine serum albumin (BSA), an analogue for bone morphogenetic protein-2.

## 2. Materials and Methods

### 2.1. Glass Synthesis

Three glass compositions, KT1, KT2 and KT3, were formulated. The control, KT1, was a SiO_2_ (0.50) + CaO (0.10) + ZnO (0.30) + Na_2_O (0.10) glass; KT2 and KT3 contain incremental additions of Ge added at the expense of Zn (Table 1) [24].

Glasses were prepared by weighing out appropriate amounts of analytical grade reagents and ball milling (1 h). Each mix was then oven dried (100 °C, 1 h) and fired (1500 °C, 1 h) in a platinum crucible and shock quenched into water. The resulting frit was dried, ground using Retsch PM 100 (Verder Scientific Inc., Newton, PA, USA) and sieved to retrieve a glass powder with a maximum particle size of <45 μm for all glasses.

#### 2.1.1. Polyacrylic Acid

Advanced Healthcare Limited (Kent, UK) supplied the PAA40 (M_W_, 35,000) and PAA200 (M_W_, 213,000). The acid was freeze-dried, ground and sieved to contain a maximum particle size of <45 μm.

#### 2.1.2. Bovine Serum Albumin

BSA (Fraction V), was purchased from Sigma Aldrich (St. Louis, MO, USA)

### 2.2. Glass Characterization

The network connectivity of the glasses was calculated using Equation (1) [25] where SiO_2_ and GeO_2_ were considered to act as network formers and CaO, ZnO and Na_2_O as network modifiers [24].

(1) $$NC=\frac{No:BOs-No:NBOs}{Total\;No:Bridging\;species}$$

where NC = Network Connectivity, BO = Bridging Oxygens, NBO = Non-Bridging Oxygens.

X-ray diffraction (XRD) patterns were collected using a PANanalytical X’Pert PRO (PANanalytical Inc., St Laurent, QC, Canada). Glass powder samples were attached to a stainless steel disc using a 20 mm glass slide. The powder compacts were then placed in the X-ray Diffractometer and scanned in the range 10° < 2θ < 80°, at scan step size 0.05° and step time of 10 s. A generator voltage of 45 kV and a tube current of 40 mA were employed using Cu kα X-ray source.

### 2.3. Scanning Electron Microscopy and Energy Dispersive X-ray Analysis

Backscattered electron (BSE) imaging was carried out with a JEOL Co. JSM-6380LV (JEOL Ltd., Tokyo, Japan) to characterize both the glasses and the fracture surfaces of cements. Compositional analysis was performed with an Energy Dispersive X-ray Analysis (EDX) Genesis Energy-Dispersive Spectrometer (JEOL Co. JSM-6380LV, JEOL Ltd., Tokyo, Japan). All EDX spectra were collected at 20 kV using a beam current of 26 nA. Quantitative EDX spectra were subsequently converted into relative concentration data.

### 2.4. Particle Size Analysis

Particle size analysis (PSA) was performed using a Coulter Ls 100 Fluid module Particle size analyzer (Beckman Coulter, Fullerton, CA, USA). The glass powder samples (*n* = 5) were evaluated in the range of 2–60 μm with a run length of 60 s. The suspension fluid used in this case was glycerol maintained at a temperature of 20–37 °C. The relevant volume statistics were calculated on each glass. The average diameters (*n* = 5) were recorded at 10%, 50%, and 90% of the cumulative volume distribution (d_10_, d_50_, and d_90_, respectively).

### 2.5. Cement Preparation

**Figure 1:**
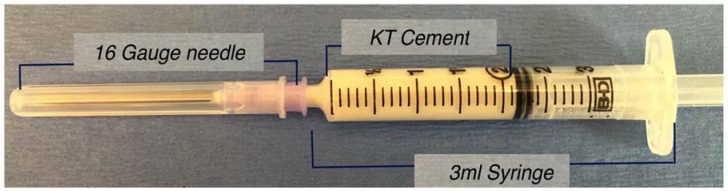
Disposable syringe with the capacity of 3 mL with an opening nozzle size of 2 mm in diameter attached with 16-gauge needle filled with KT cement.

Two techniques of cement preparation were employed. The first technique involved mixing by hand (h) using a spatula and a clean glass slab and the second technique involved injection (i) where a spatula was again used to mix the cement which was then placed in a disposable syringe (capacity, 3 mL) with an opening nozzle size of 2 mm diameter attached onto a 16 gauge needle (Figure 1).

The cements were formulated in a powder:liquid (P:L) ratio of 1:0.75, i.e., 1 g of glass powder (<45 μm) was mixed with 0.375 g PAA and 0.375 mL of liquid. The liquid portion was 100% double de-ionized (DDI) water with three different loads of standard protein BSA. Table 2 outlines the formulations tested.

### 2.6. Handling Characteristics

#### 2.6.1. Working Time

The cement working time (T_W_), measured in ambient temperatures using a stopwatch, was defined as the period of time from the start of mixing during which it was possible to manipulate the material without having an adverse effect on its properties [26]. The (h) technique mixed powder and liquid components with a spatula and evaluated the T_W_ in line with the above definition while the (i) technique involved mixing both components with the spatula for the first 30 s, then depositing the resultant cement in the disposable syringe and injecting it back onto the glass plate where, again, T_W_ was evaluated in line with the above definition.

#### 2.6.2. Setting Time Measurement and the Preparation of GPCs

##### 2.6.2.1. Setting Time Measurement

Setting times (T_S_) of these cements were measured according to the ISO9917 standard for dental cements [27] using a Gillmore apparatus (Humboldt Mfg. Co, Elgin, IL, USA) [27]. For the (h) technique, the mould was filled using a spatula, while, for the (i) technique, the mould was filled using the cement injected from the syringe. In line with the ISO standard, a sample was considered set when a 400 g mass loaded to a needle with a tip diameter of 1 mm did not make any visible impression on the surface of the sample in 100% relative humidity at 37 °C.

##### 2.6.2.2. Preparation of GPCs

Injectability of the cement was evaluated using a disposable syringe that extruded the cements through the syringe using hand pressure. Each individual syringe was filled with 3 g of mixed cement after 30 s of mixing; the syringes were then filled using a spatula. For example, the first syringe extruded the cement as soon as it was filled, the second syringe extruded the cement 5 min after the filling time, the third syringe extruded the cement 10 min after the filling time; this process continued for the remaining syringes in 5 min increments until hand pressure was unable to inject any further cement. The weight of the injected cement was then measured and injectability was calculated using the following Equation (2) [28]:

(2) $$\mathrm{Injectability}\left(\%\right)=\frac{\left(\mathrm{Cement}\;\mathrm{weight}\;\mathrm{expelled}\;\mathrm{from}\;\mathrm{the}\;\mathrm{syringe}\right)}{\left(\mathrm{Total}\;\mathrm{cement}\;\mathrm{weight}\;\mathrm{before}\;\mathrm{injection}\right)}$$

### 2.7. Mechanical Properties

#### 2.7.1. Compressive Strength Measurement

**Figure 2:**
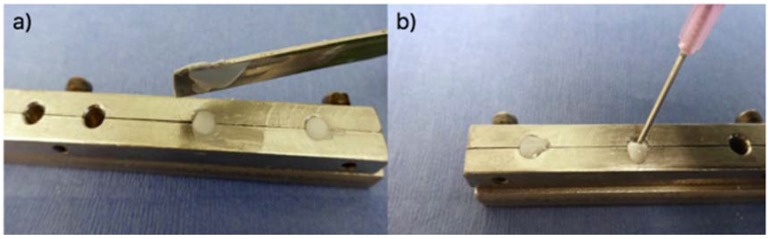
The preparation of the compressive strength samples. (**a**) hand technique (h); (**b**) injection technique (i).

The compressive strength (σc) of five cement samples of each formulation were evaluated in ambient air (23 ± 1 °C) according to ISO9917 [27]. Samples were tested after 1, 7 and 30 days using a computer controlled Instron Universal Testing System (Instron Corp, Norwood, MA, USA) fitted with a ±2 KN load cell at a crosshead speed of 1 mm·min^−1^. In accordance with ISO9917 [27], freshly mixed cement was used to fill the moulds (4 mm·Ø by 6 mm height) using two different techniques. The first set of moulds were filled using the (h) technique with a spatula (Figure 2a) and the second set of moulds were filled with freshly mixed cement injected from the syringe (the (i) technique) to excess and then covered with acetate sheet (Figure 2b).

The mould/cement/acetate constructs were then sandwiched between two stainless steel plates, and the constructs clamped and incubated (37 °C, 1 h). Following incubation, samples were unclamped and excess flash around the moulds was removed using 1200-grit SiC paper. Once ground, the samples were de-moulded, placed in distilled water and incubated in DDI water (37 °C) for 1, 7 and 30 days. σc was calculated according to Equation (3) [27]:

(3) $$\sigma c=\frac{4\rho }{\pi d^{2}}$$

where *ρ* = maximum applied load (N), *d* = diameter of sample (mm).

#### 2.7.2. Biaxial Flexural Strength

**Figure 3:**
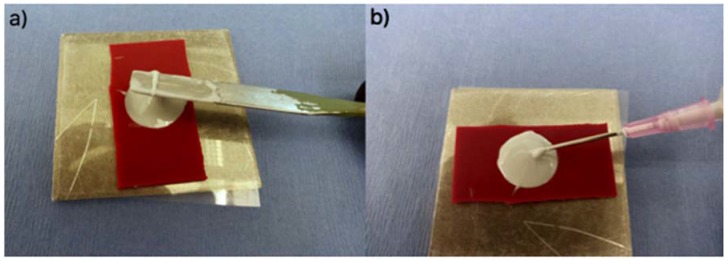
The preparation of the Biaxial Flexural Strength samples. (**a**) hand technique (h); (**b**) injection technique (i).

Sixty seconds after mixing commenced (*n* = 5 samples for each cement tested at 1, 7 and 30 days), rubber moulds (8 mm·Ø, 2 mm thick) were filled with cement by hand (Figure 3a) using a spatula and an equal number of moulds were also filled with cement injected from a syringe (Figure 3b) to excess with cement and placed between 2 stainless steel plates, clamped, and incubated (37 °C, 1 h).

Each of the samples (*n* = 5) were subsequently de-moulded and incubated in DDI water for 1, 7 and 30 days for both the (h) and (i) technique. The biaxial flexural strength (σf) of the cements was determined in a similar fashion to that used by Williams et al. [29] which considered three support bearings on the test jig fixed to an Instron Universal Testing Systems (Instron Corp, Norwood, MA, USA) apparatus using a load cell of 1 kN. Testing was performed at a crosshead speed of 1 mm·min^−1^. Five samples for each cement formulation and incubation time were tested. σf was calculated according to Equation (4) [29].

(4) $$BFs=\frac{\rho \left(N\right)}{t^{2}}\left\{0.63In\left(\frac{r}{t}\right)+1.156\right\}$$

where *r* = fracture load (*N*), *t* = sample thickness (mm), *r* = radius of the support diameter (mm).

### 2.8. Ion and Bovine Serum Albumin Release Profile

The concentrations of Si, Ca, Zn, Na and Ge ions released from the cements for 1, 7 and 30 days were determined by analyzing the water extracts in which samples of each set cement were stored using a Perkin Elmer Atomic Absorption Spectrometer 800 (AAS800, Perkin Elmer, Waltham, MA, USA). Then, 10 mL aliquots of DDI water were kept at 37 °C in lidded containers. Samples (*n* = 5) of each cement (8 mm·Ø, 2 mm thick) were then stored for 1, 7 and 30 days in water. Following removal of cement samples from their aliquots, a 1:10 dilution of the storage water was made using purified water. Calibration standards for Si, Ca, Zn, Na and Ge elements were prepared from a stock solution on a gravimetric basis. Five target calibration standards were prepared for each ion with 0.1, 0.3, 0.5, 0.7 and 1.0 part per million (ppm) concentrations with DDI water used as a blank. Samples for Ca, Zn, Na and Ge ion analysis were diluted in a ratio of 1:10; that is, each 1 mL of concentrated sample was mixed with 10 mL of DDI water while samples for Si analysis were diluted in a ratio of 1:30. A pilot study was conducted to determine the appropriate ratio for dilution of all elements. 

Glass polyalkenoate cement (GPC) samples were loaded with 3%, 6% and 9% of BSA (*n* = 5) and were placed into an Eppendorf tube containing 10 mL of DDI water for 1, 7 and 15 days for each load in order to determine the release of the BSA. The water was collected and stored at 2–8 °C until ready to be assayed. Protein concentration was measured using the Pierce Coomassie Plus Assay Kit according to the manufacturer’s instructions (Thermo Fisher, IL, USA). Following incubation of sample aliquots with the dye, the absorbance at 595 nm was measured using VersaMax plate reader (Molecular Devices, Sunnyvale, CA, USA). The protein concentration was determined by comparison to a standard curve produced using BSA.

### 2.9. Statistical Analysis

One-way analysis of variance (ANOVA) was used to analyze the data for handling and mechanical properties using non-parametric Kruskal-Wallis. A post hoc Bonferroni test was used to compare the relative means and to report the statistically significant differences when *p ≤* 0.05. Statistical analysis was performed using SPSS software (IBM SPSS statistics 21, IBM Corp., Armonk, NY, USA).

## 3. Results and Discussion

Glass polyalkenoate cements (GPCs) made by the (h) method and IGPCs by the (i) method were formulated as outlined above and then evaluated both physically and mechanically.

### 3.1. Glass Characterization

**Figure 4:**
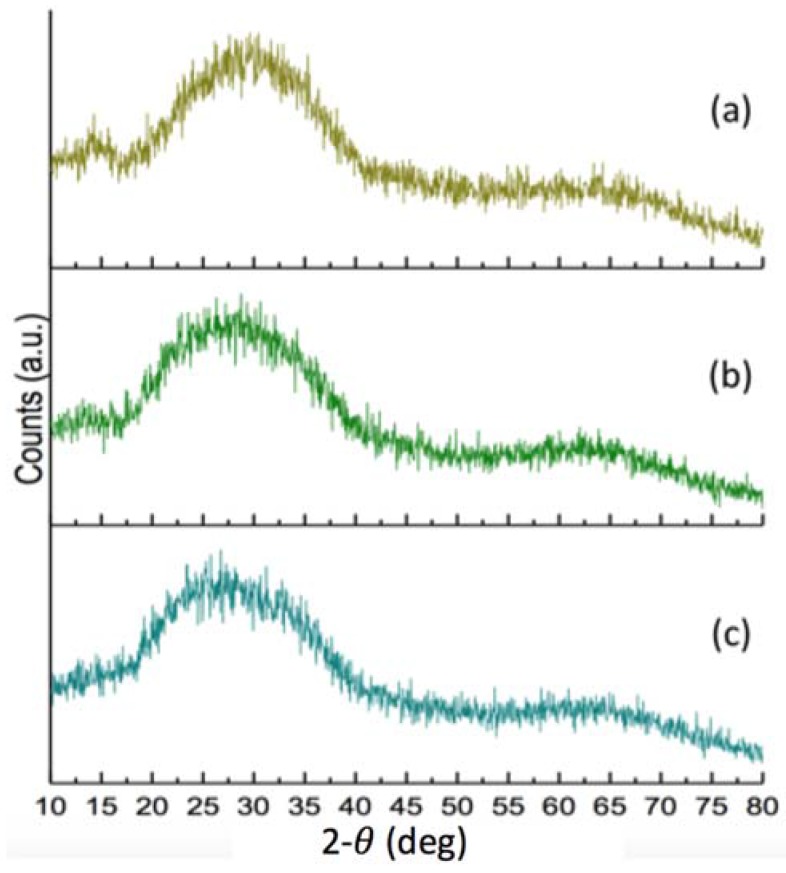
X-ray diffraction (XRD) patterns of the formulated glasses (KT) series confirming all glasses were fully amorphous. (**a**) KT1; (**b**) KT2 and (**c**) KT3.

The network connectivity for KT1, KT2 and KT3 was calculated to be 2, 2.2 and 2.4, respectively [24], as increasing the amount of GeO increased network connectivity. XRD confirmed that no crystalline phases were present within the glasses (Figure 4).

### 3.2. Scanning Electron Microscopy and Energy Dispersive X-ray Analysis Particle Size Analysis 

**Figure 5:**
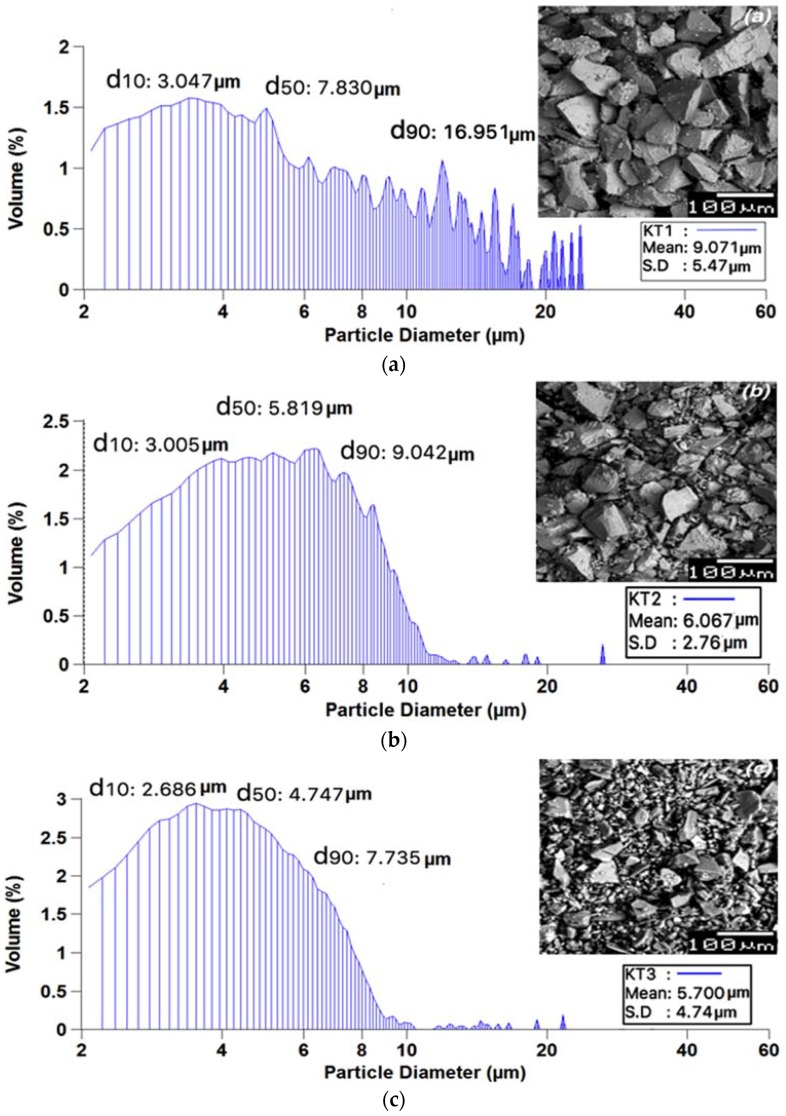
Particle size analysis (PSA) and scanning electron microscopy (SEM) micrographs. (**a**) KT1; (**b**) KT2; (**c**) KT3. S.D: standard deviation.

Each glass was examined by. Scanning electron microscopy (SEM) (Figure 5a–c). The KT1 (a), KT2 (b) and KT3 (c) glasses all had different particle size distributions, with the mean particle size for KT1 being 9 μm, 6 μm for KT2 and 5 μm for KT3. This would be an artifact of the action of grinding which is difficult to control to the extent that particle sizes are standardized. The smaller particles increase the overall surface area available for reaction, which contributes to a more rapid set [30]. SEM images also record this variation in particle size.

Energy dispersive X-ray (EDX) was performed during microscopy and confirmed that the ion contents incorporated in the oxide reagents were present in comparable amounts in the fired glasses (Table 3).

### 3.3. The Influence of GeO Incorporation and Acid Concentration on Working and Setting Times Measurement of GPCs and Injectability of the Cements

**Figure 6:**
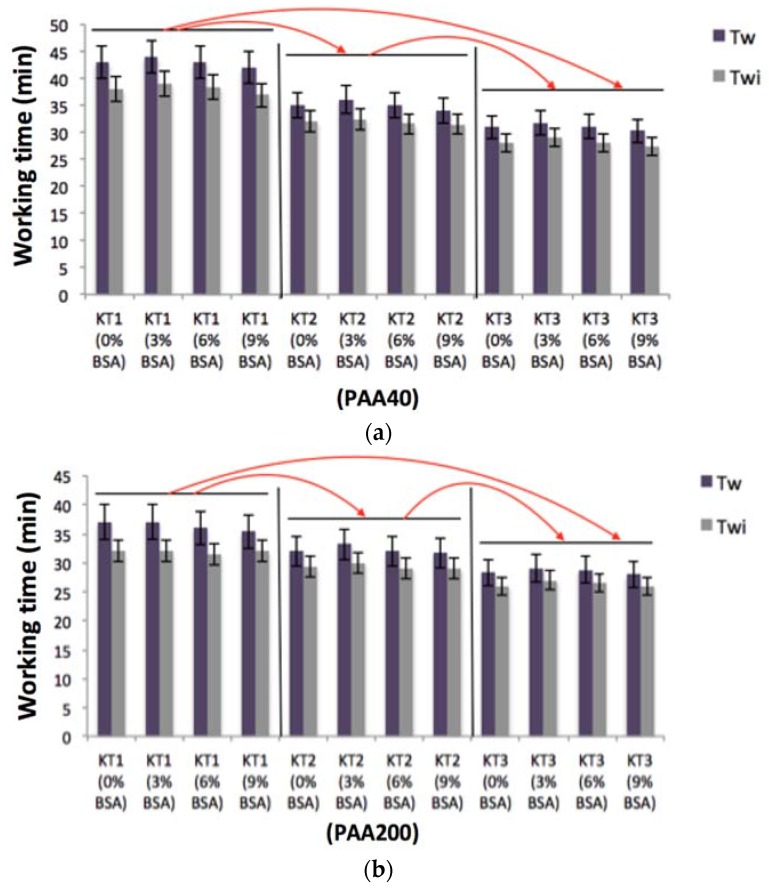
(**a**) Working times (T_W_) of the cement series and working times of the injectable cement (T_Wi_) with PAA40; (**b**) T_W_ and T_Wi_ with PAA200. Red arrows show statistical significance (*p* < 0.05) between the cement groups.

**Figure 7:**
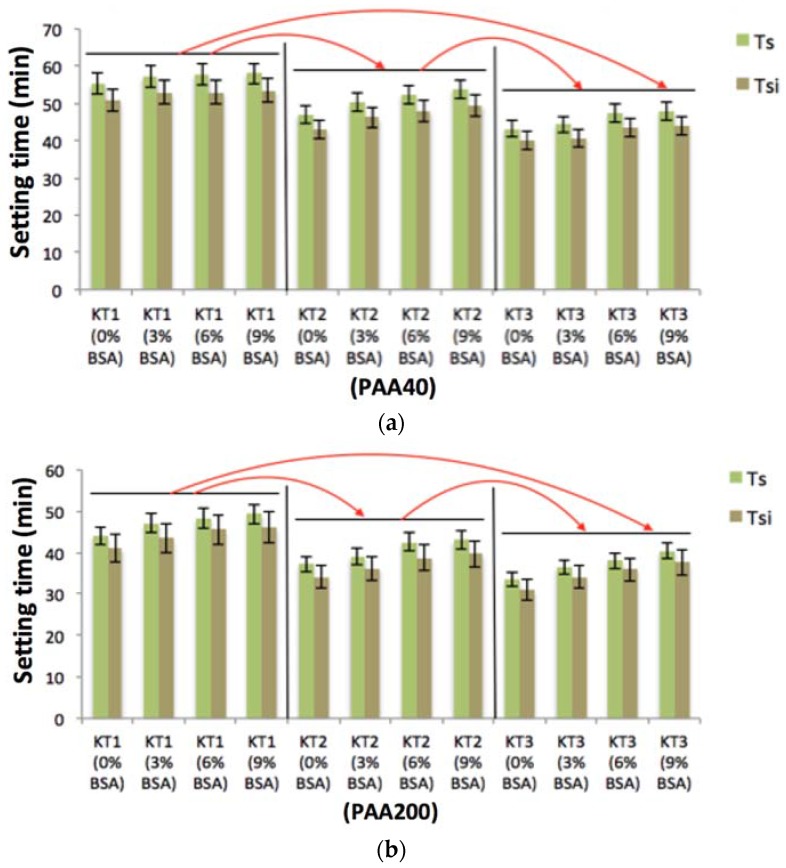
(**a**) Setting times (Ts) of the cement series and setting times of the injectable cement (Tsi) with PAA40; (**b**) Ts and Tsi with PAA200. Red arrows show statistical significance (*p* < 0.05) between the cements groups.

When incorporated at up to 9 wt %, BSA incorporation did not significantly influence the handling properties of the cements. However, there were differences in handling properties as a result of Ge incorporation in the glass phase. Figure 6a,b displays the T_W_ of the cements. T_W_ of the cements based on PAA40 linearly decreased from 42 min to 33 min with increasing Ge content and from 37 min to 29 min when mixed with PAA200. Figure 7a,b displays the Ts of the cements. Due to the increase of Ge in the glasses, Ts decreased from 57 min for KT1 to 45 min for KT3 when the glasses were mixed with PAA40 and from 47 min for KT1 to 35 min for KT3 when mixed with PAA200. All of these results show a statistically significant decrease (*p* < 0.05) as a result of Ge incorporation. 

The decreasing trends experienced in both T_W_ and Ts can be explained by the resultant increase in susceptibility to acid attack as increased amounts of Ge increase network connectivity (NC), resulting in the glasses releasing more cations into the environment thereby increasing carboxylic (COO−)/metal cation bonding rates of ion gelation [31]. The introduction of Ge ions, which have a 4+ charge, may also increase the bonding rate of the un-bonded COO-molecular chains [32].

**Figure 8:**
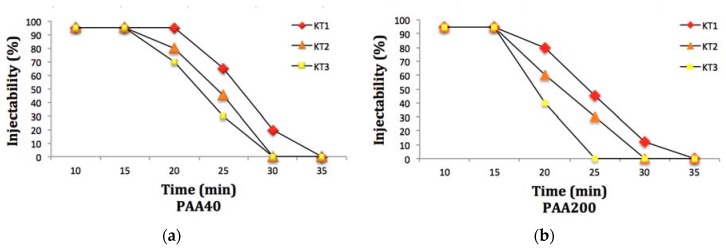
Injectability of cements depending on the time after mixing (**a**) PAA40 and (**b**) PAA200.

The injectability of the cements were evaluated using Equation (2). In PAA40 (Figure 8a) the KT1 IGPC maintained 97% injectability after 20 min (once the 3 mL syringe had extruded all the cement, around 3% of the cement remained in the 16 gauge needle), but this iteration of cement was no longer injectable after 35 min. KT2 and KT3 maintained around 97% injectability after 15 min but were no longer injectable after 30 min. With cements incorporating PAA200, the KT1, KT2 and KT3 IGPCs maintained 97% injectability after 15 min; however, they were no longer injectable after 25–35 min, as shown in Figure 8b. The difference in injectability between the three cements could be due to the increase in molecular weight of the acid (Mw). However, the T_W_ and Ts decreased in KT2 and KT3 with the addition of Ge as previously explained. Therefore, the amount of extruded injectable cements was found to decrease with the increased amount of Ge.

### 3.4. The Influence of GeO, BSA Incorporation and Acid Concentration on Compressive (σc) and Biaxial Flexural (σf) Strengths of KT Cements

**Figure 9:**
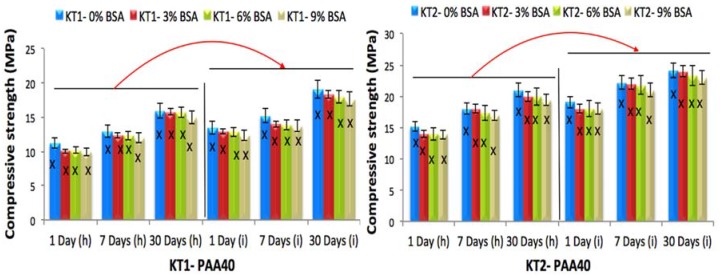

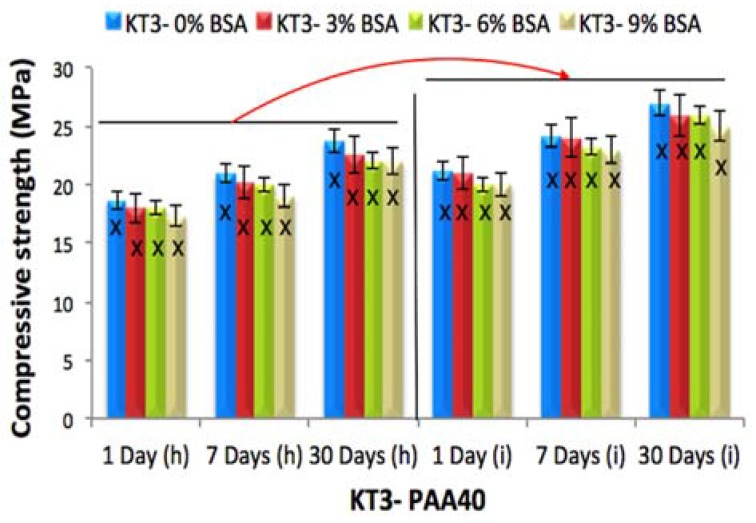
Compressive strength for the cement series over 1, 7 and 30 days maturation with PAA40, (h) the hand version and (i) the injection version, error bars represent SD, X and red arrows (between (h) and (i) version groups) show statistically significant difference (*p* < 0.05).

**Figure 10:**
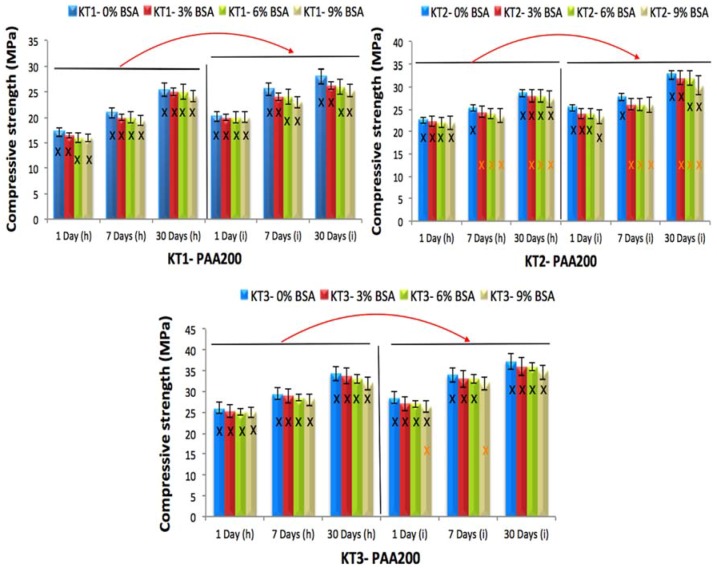
Compressive strength for the cement series over 1, 7 and 30 days maturation with PAA200, (h) the hand version and (i) the injection version, error bars represent SD, X and red arrows (between (h) and (i) version groups) show statistically significant difference (*p* < 0.05).

The compressive strength (σc) was evaluated according to ISO9917 [27]. Samples were submerged in DDI water for 1, 7 and 30 days prior to testing using both the (h) and (i) techniques. Figure 9 and Figure 10 display the change of σc with time for all cement formulations, which was found to increase with maturation for each technique as well as with acid molecular weight, with the largest increase occurring between 7 and 30 days. The incorporation of Ge in the KT glass phase also resulted in increased σc of the cements, with statistical differences being recorded between the KT1, KT2 and KT3 cements at 1, 7, and 30 days maturation. KT cements acted as expected, where increased PAA concentration resulted in increased strength due to the higher concentration of COO-groups available for forming ionic cross-bridges within the cement matrix [33].

Cements delivered by the (i) technique exhibited higher strengths than those delivered by the (h) technique, which could be due to the cement being injected from the 16-gauge needle, resulting in lower porosity since the cement is extruded closely together it reduces the amount of air in the cement. The BSA did not significantly influence the strengths of cements (*p <* 0.05) when incorporated. 

**Figure 11:**
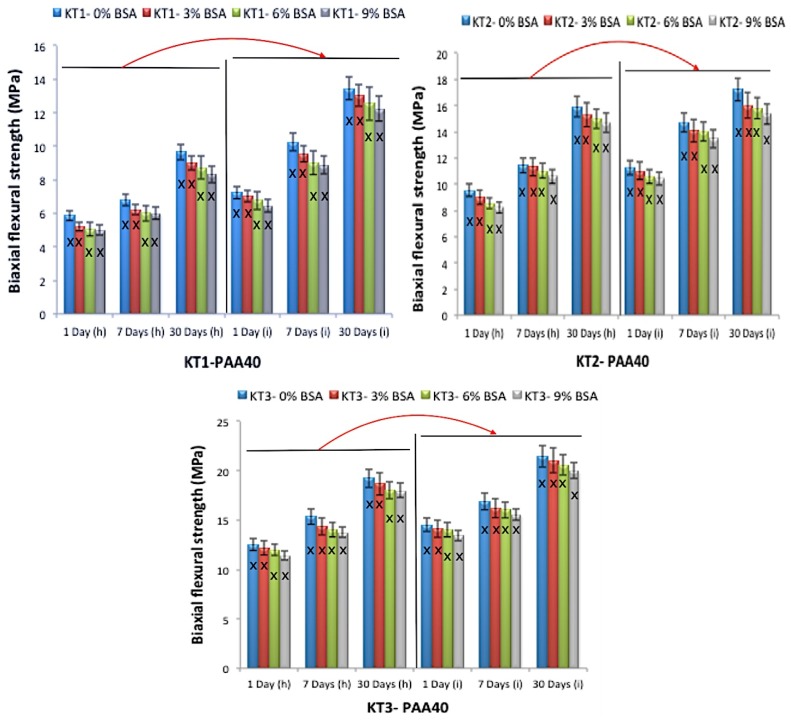
Biaxial flexural strength for the cement series over 1, 7 and 30 days maturation with PAA40, (h) the hand version and (i) the injection version, error bars represent SD, X and red arrows (between (h) and (i) version groups) show statistically significant difference (*p* < 0.05).

**Figure 12:**
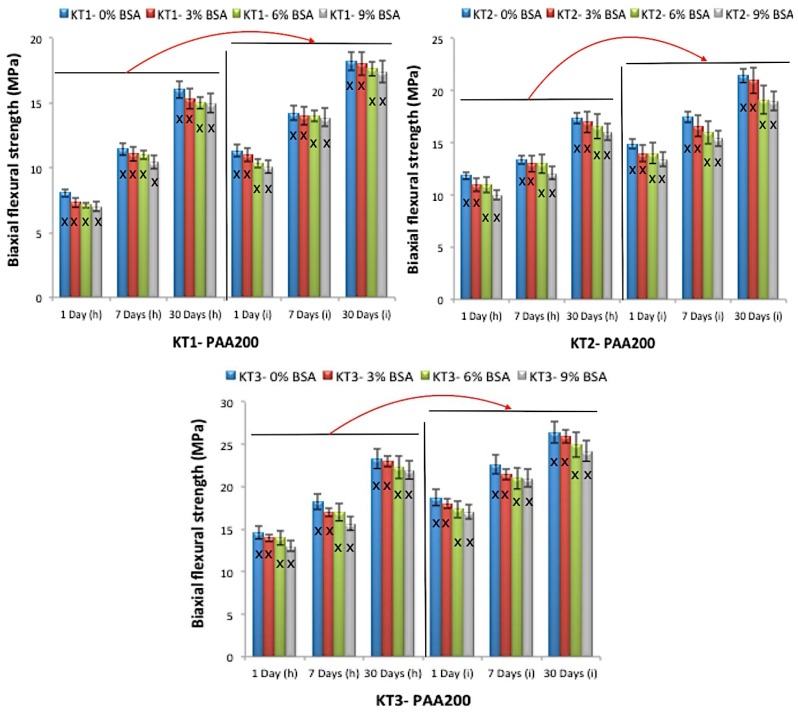
Biaxial flexural strength for the cement series over 1, 7 and 30 days maturation with PAA200, (h) the hand version and (i) the injection version, error bars represent SD, X and red arrows (between (h) and (i) version groups) show statistically significant difference (*p* < 0.05).

Flexural strength testing was conducted using a method similar to that used by Williams et al. [29]. Figure 11 and Figure 12 present the σf results; changes in strength were evident with the addition of Ge in respect to time, with similar trends to those seen with the compressive tests with respect to maturation.

Strength increases over time (maturation) as a result of the ions released from the glass chelating with the COO- from the acid component [34,35,36,37], through a continuous acid-base reaction [33]. In this study, in addition to maturation, strengths also increased with Ge content. Ge is a 4+ valency ion; thus, it will bond and increase the network connectivity (NC) of the cement [24]. Hill et al. [38] described the entanglement of polyanion chains during GPC setting and how they limit lateral movement, while interactions with neighboring chains limit longitudinal movement. Thus, it is probable that relations between multivalent Ge^4+^ ions, or complexes thereof, interrelate with more than two polyanions to increase chain bonding and entanglement, thus creating stronger cements [39,40,41].

**Figure 13:**
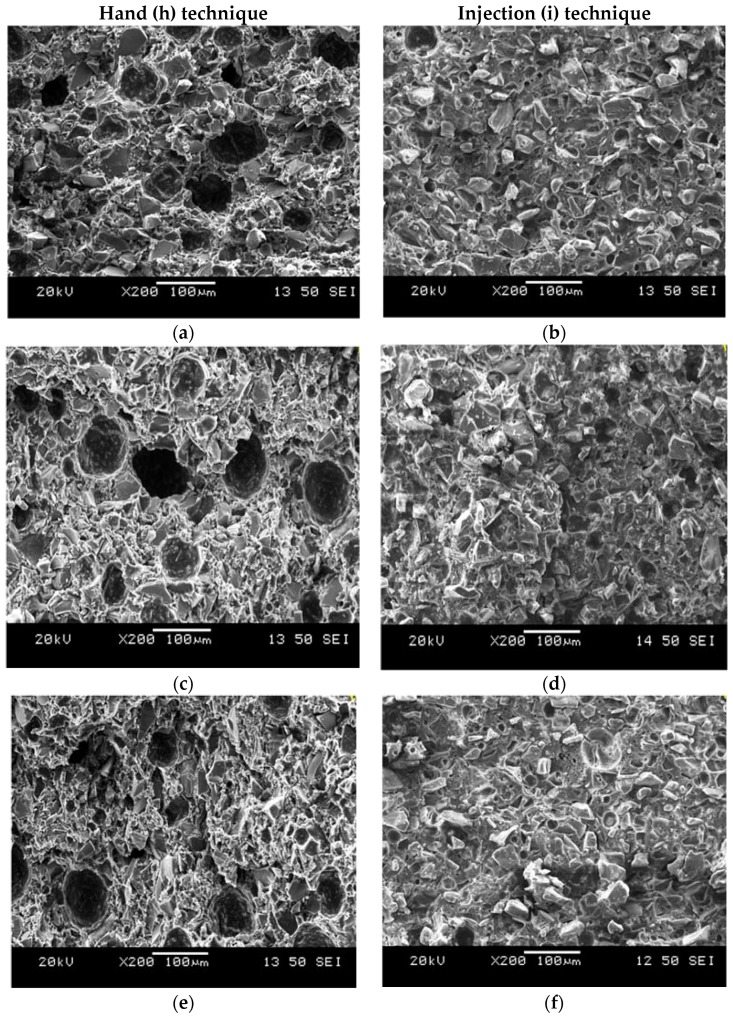
SEM images of the fracture surface of KT1 (**a**,**b**); KT2 (**c**,**d**) and KT3 (**e**,**f**) cements after 30 days with PAA200 for (h) and (i) technique.

Bovine serum albumin (BSA) incorporation did not affect handling properties or strength. However, there was a significant difference between cements delivered by the (h) technique and the (i) technique. Cements delivered by the (i) technique were stronger, possibly because the pressure that was applied while extruding the cement from the narrow 16-gauge needle had decreased the amount of porosity within the cements (Figure 13). This can be explained in such a way that when performing the (h) technique using the spatula, there is no pressure applied to the cement, in turn allowing the air to remain inside the cements. Additionally, when mixing the GPC with the spatula, it is possible that more air was introduced into the cement; on the other hand, when performing the (i) technique using the 16-gauge needle, the cement is extruded with hand pressure through the narrow needle, therefore forcing the cement to be more close together, in turn reducing the pores in the cement as well as increasing the strength [42].

### 3.5. Morphology of Cement Fracture Surfaces

Figure 13a–f shows the fracture surfaces of cements delivered by both the (h) and (i) techniques; KT1 (a, b) and KT2 (c, d) and KT3 (e, f) after 30 days maturation for the PAA200 σc test. The fracture surfaces of the cement produced by the (i) technique contained less residual pores than the (h) technique, as can be observed from low magnification images. Residual pores of different sizes remained in both techniques but some of the porosity in the (h) technique were less cohesive than the (i) technique (Figure 13), the (i) technique will not allow hollow zones to form within the cement, thus increasing the cements cohesiveness, as the pores in the (h) technique are larger. Therefore, the porosity in the cement affects recorded strengths, as bigger pores in the cement will have a deleterious effect on strength [42] by creating hollow zones within the cements, resulting in the cements having lower strengths, as shown previously in Figure 10.

### 3.6. Ion and Bovine Serum Albumin Release Profile

**Figure 14:**
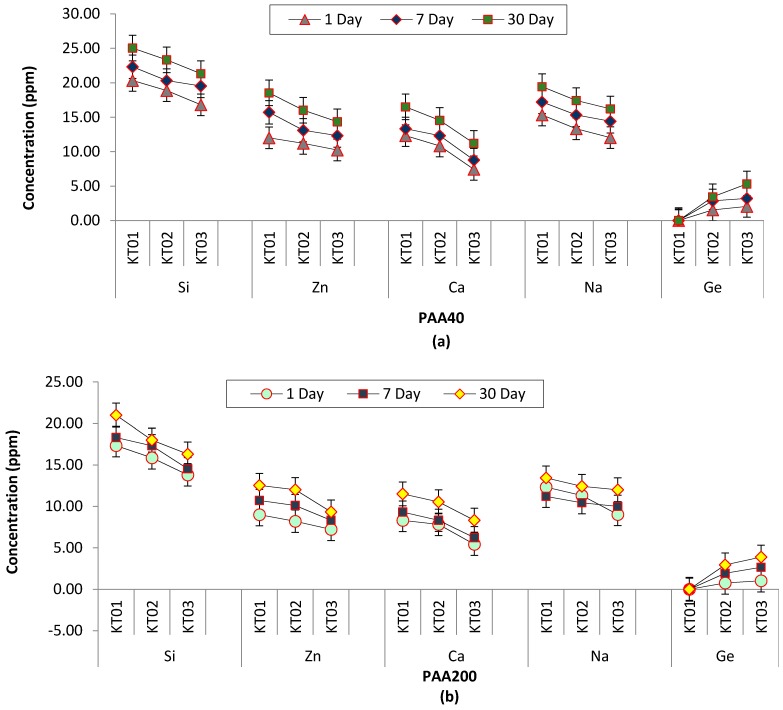
Calculated results for ion release of the (i) technique over 1, 7 and 30 days. (**a**) The average for each glass with PAA40; (**b**) The average for each glass with PAA200. Error bars represent the SD.

Na, Sr, Si, Zn, Ca and Ge ions released from the cements were measured cumulatively over 1, 7 and 30 days for the samples produced by the (i) technique. Figure 14a,b exhibits the Na, Sr, Si, Zn, Ca and Ge ion release profiles. The average ion concentration for each cement has been calculated, including with respect to BSA loading. As would be expected, all the ions released increased in number with maturation in any glass composition. However, the incorporation of Ge into the glass resulted in reduced ion release for the Na, Sr, Si, Zn and Ca ions at the same time point, with the obvious exception of Ge, which, understandably, increases in line with its content in the precursor glass from which it elutes.

Unsurprisingly, incorporating increasing amounts of Ge in the glass phase resulted in increasing amounts of Ge ions released from the resultant cements. There is also less Zn ion release from the cements going through the series as the increasing Ge content is as a result of a reduced Zn content. However, the quantity of all other ions released from the cements also decreased with increased Ge content, suggesting that Ge increased the glass network, moreover the network connectivity will expand and will increase the NBOs in the glass, in turn causing a decrease in ion released from the cement. As the mechanical properties of the cements increase with increasing Ge content it suggests that it is the Ge ion, with its 4+ valency, that is dominating the cement setting reaction.

**Figure 15:**
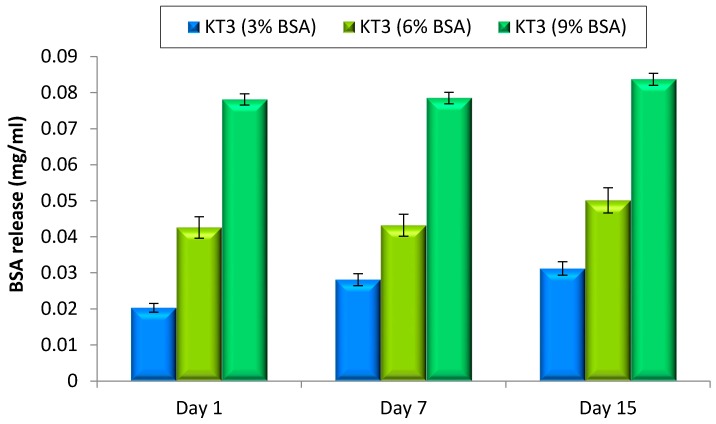
BSA release profiles for KT3 (i) technique containing BSA of 3 loads (3%, 6% and 9%) in mg/mL. Error bars represent SD.

Glass polyalkenoate cements GPCs have been shown to have the ability to be used as a carrier for BSA (used as a model protein). Figure 15 presents the results of the BSA release from the best performing cement (KT3) for 1, 7 and 15 days. The release of BSA was found to increase with maturation time, although the most rapid release occurred over the first day. This release of BSA is likely due to diffusion from the unset surface of the GPC due to the elongation of setting time. As expected, increasing the BSA load resulted in an increase in the amount of BSA released. On the other hand, during this study results showed no significant effect on handling and mechanical properties with the incorporation of BSA into GPCs.

## 4. Conclusions

In this study, GPCs have been synthesized using a Ge-containing glass as the powder component and PAA, DDI water and BSA as the liquid component, and these have been shown to be injectable (97% up to 15 min); Ge incorporation caused an increase in the mechanical properties and a decrease in handling properties. This could be due to the smaller sized pores of the cement that resulted from using the (i) technique, which caused the cement to be stronger, therefore a decrease in the size and amount of pores increases the mechanical properties [42]. The addition of Ge in KT2 and KT3 increases the NC of the glass and allows the bonding of more ions with the cement. Also, the GPCs were found to be potential carriers for bone morphogenetic proteins as there was no significant difference when adding different loadings of BSA. Additional work is ongoing to assess the effect of KT cement series on the bonding and biological properties for fracture fixation and stabilization.

## Figures and Tables

**Table 1:** KT glass compositions (Mol.%) [24].

Composition	SiO_2_	CaO	ZnO	Na_2_O	GeO_2_
*KT1*	0.50	0.10	0.30	0.10	0
*KT2*	0.50	0.10	0.27	0.10	0.03
*KT3*	0.50	0.10	0.24	0.10	0.06

**Table 2:** Formulation table showing the changing liquid portion of the cement samples (ratio 1:0.75).

Cement Formulations	Powder Components	Liquid Components
Name	GPCs (g)	PAA40 and PAA200 (wt %) DDI Water (wt %) BSA (wt %)
KT1	1 g	0.375/0.375 0.375 0, 3, 6, 9
KT2	1 g	0.375/0.375 0.375 0, 3, 6, 9
KT3	1 g	0.375/0.375 0.375 0, 3, 6, 9

GPC: Glass polyalkenoate cement; PAA: Polyalkenoic acid; DDI: Double de-ionized; BSA: Bovine serum albumin.

**Table 3:** Composition in wt % as verified by energy dispersive X-ray (EDX).

Composition	KT1	KT2	KT3
Si	31.3	29.7	28.2
Ca	8.8	8.5	7.8
Zn	26.5	22.7	22.1
Na	8.9	8.3	7.6
Ge	-	2.2	5.2

## References

[1] Qui Y., Hamilton S.K., Temenoff J. (2011). Improving mechanical properties of injectable polymers and composites. Inject. Biomater. Sci. Appl..

[2] Laurencin C.T., Ambrosio A.M.A., Borden M.D., Cooper J.A. (1999). Tissue Engineering: Orthopedic Applications. Annu. Rev. Biomed. Eng..

[3] Chow C.L., Takagi S. (2001). A natural bone cement—A laboratory novelty led to the development of revolutionary new biomaterials. J. Res. Natl. Inst. Tech..

[4] Wren A.W. (2008). Strontium Substituted Glass Ionomer Cements for Skeletal Applications. Ph.D. Thesis.

[5] Wang X., May J., Wang Y., He B. (2002). Bone repair in radii and tibias of rabbits with phosphorylated chitosan reinforced calcium phosphate cements. Biomaterials.

[6] Higgins T.F., Dodds S.D., Wolfe S.W. (2002). A biomechanical analysis of fixation of intra-articular distal radial fractures with calcium phosphate bone cement. J. Bone Jt. Surg. Am. Vol..

[7] Hidaka N., Yamano Y., Kadoya Y., Nishimura N. (2002). Calcium phosphate bone cement for treatment of distal radius fractures: A preliminary report. J. Orthop. Sci..

[8] Aoki H., Ukegawa Y., Yamamuro T., Onishi H. (1992). Ceramic biomaterial. Manual of Orthopaedic Materials.

[9] Niwa S., Yamamoto H. (1998). Biological reaction and clinical application of calcium phosphate cement. Kansetugeka. J. Jt. Surg..

[10] Khairoun I., Driessens F.C., Boltong M.G., Planell J.A., Wenz R. (1999). Addition of cohesion promotors to calcium phosphate cements. Biomaterials.

[11] Cattani-Lorente M.A., Godin C., Meyer J.M. (1993). Early strength of glass ionomer cements. Dent. Mater..

[12] Nicholson J.W. (1998). Adhesive dental materials–A review. Int. J. Adhes. Adhes..

[13] Zimehl R., Hannig M. (2000). Non metallic restorative materials based on glass ionomer cements-recent trends and developments. Colloids Surf. A.

[14] Nicholson J.W. (1998). Chemistry of glass-ionomer cements: A review. Biomaterials.

[15] Towler M.R., Kenny S., Boyd D., Pembroke T., Buggy M., Hill R.G. (2004). Zinc ion release from novel hard tissue biomaterials. Biomed. Mater. Eng..

[16] Endotherapeutics: Serenocem Ear Cement and Granules. http://www.endotherapeutics.com.au/serenocem.

[17] Invotec International^®^: Serenocem^TM^ Ostologic Cement. http://www.mundinc.com/uploads/serenocem_otologic_cement.pdf.

[18] Wren A.W., Kidari A., Cummins N.M., Towler M.R. (2010). A spectroscopic investigation into the setting and mechanical properties of titanium containing glass polyalkenoate cements. J. Mater. Sci. Mater. Med..

[19] Sawai J. (2003). Quantitative evaluation of antibacterial activities of metallic oxide powders (ZnO, MgO and CaO) by conductimetric assay. J. Microbiol. Methods.

[20] Catelan A., Padilha A.C., Salzedas L.M., Coclete G.A., dos Santos P.H. (2008). Effect of radiotherapy on the radiopacity and flexural strength of a composite resin. Acta Odontol. Latinoam..

[21] Boyd D., Towler M.R., Watts S., Hill R.G., Wren A.W., Clarkin O.M. (2008). The role of Sr^2+^ on the structure and reactivity of SrO-CaO-ZnO-SiO_2_ ionomer glasses. J. Mater. Sci. Mater. Med..

[22] Clarkin O., Boyd D., Towler M.R. (2009). Comparison of failure mechanisms for cements used in skeletal luting applications. J. Mater. Sci. Mater. Med..

[23] Varshneya A.K. (1994). Fundamentals of Inorganic Glasses.

[24] Khader B.A., Curran D.J., Peel S., Towler M.R. (2016). Glass Polyalkenoate Cements Designed for Cranioplasty Applications: An Evaluation of Their Physical and Mechanical Properties. J. Funct. Biomater..

[25] Towler M.R., Stanton K.T., Mooney P., Hill R.G., Moreno N., Querol X. (2002). Modelling of the glass phase in fly ashes using network connectivity theory. J. Chem. Technol. Biotechnol..

[26] (2007). Dentistry—Water-Based Cements-Part 1: Powder/Liquid Acid-Base Cements, ISO 9917-1.

[27] (1991). Dentistry—Water-Based Cements-Part 1: Powder/Liquid Acid-Base Cements, ISO 9917.

[28] Khairoun I., Boltong M.G., Driessens F.C., Planell J.A. (1998). Some factors controlling the injectability of calcium phosphate bone cements. J. Mater. Sci. Mater. Med..

[29] Williams J.A., Billington R.W., Pearson G.J. (2002). The effect of the disc support system on biaxial tensile strength of a glass ionomer cement. Dent. Mater..

[30] Prentice L.H., Tyas M.J., Burrow M.F. (2005). The effect of particle size distribution on an experimental glass-ionomer cement. Dent. Mater..

[31] Wren A.W., Hansen J.P., Hayakawa S., Towler M.R. (2013). Aluminum-free glass polyalkenoate cements: Ion release and in vitro antibacterial efficacy. J. Mater. Sci. Mater. Med..

[32] Dickey B.T., Kehoe S., Boyd D. (2013). Novel adaptations to zinc-silicate glass polyalkenoate cements: The unexpected influences of germanium based glasses on handling characteristics and mechanical properties. J. Mech. Behav. Biomed. Mater..

[33] Wilson A.D., Nicholson J.W., West A.R., Baxter H. (1993). Acid-Base Cements: Their Biomedical and Industrial Applications (Chemistry of Solid State Materials).

[34] Wren A.W., Coughlan A., Palcek L., Towler M.R. (2012). Gallium containing glass polyalkenoate anti-cancerous bone cements: Glass characterization and physical properties. J. Mater. Sci. Mater. Med..

[35] Boyd D., Towler M.R. (2005). The processing, mechanical properties and bioactivity of zinc based glass ionomer cements. J. Mater. Sci. Mater. Med..

[36] De Barra E., Hill R.G. (1998). Influence of alkali metal ions on the fracture properties of glass polyalkenoate (ionomer) cements. Biomaterials.

[37] Clarkin O., Boyd D., Towler M.R. (2010). Strontium-based glass polyalkenoate cements for luting applications in the skeleton. J. Biomater. Appl..

[38] Hill R.G. (1993). The fracture properties of glass polyalkenoate cements as a function of cement age. J. Mater. Sci..

[39] Barkema G.T., Panja D., van Leeuwen J.M.J. (2011). Structural modes of a polymer in the repton model. J. Chem. Phys..

[40] Rubinstein M. (1987). Discretized model of entangled-polymer dynamics. Phys. Rev. Lett..

[41] McLeish T.C.B. (2002). Tube theory of entangled polymer dynamics. Adv. Phys..

[42] Pourbeik P., Beaudoin J.J., Alizadeh A.R., Raki L. (2013). Mechanical property-porosity relationships of layered calcium silicate hydrate phases. Mater. Struct..

